# Upconversion Nanophosphor-Involved Molecularly Imprinted Fluorescent Polymers for Sensitive and Specific Recognition of Sterigmatocystin

**DOI:** 10.3390/polym9070299

**Published:** 2017-07-22

**Authors:** Jing-Min Liu, Feng-Zhen Cao, Guo-Zhen Fang, Shuo Wang

**Affiliations:** 1 Beijing Advanced Innovation Center for Food Nutrition and Human Health, Beijing Technology & Business University (BTBU), Beijing 100048, China; liujingmin@nankai.edu.cn; 2 School of Medicine, Nankai University, Tianjin 300071, China; 3 Key Laboratory of Food Nutrition and Safety, Ministry of Education, Tianjin University of Science and Technology, Tianjin 300457, China; nkbluer@163.com (F.-Z.C.); fangguozhen@tust.edu.cn (G.-Z.F.)

**Keywords:** sterigmatocystin (ST), upconversion nanophosphors, molecularly imprinted polymers, fluorescence detection

## Abstract

Originated from the bottom-up synthetic strategy, molecularly imprinted polymers (MIPs) possess the inherent ability of selective and specific recognition and binding of the target analytes, with their structural cavities that can match the target molecules in respect to size, shape, and functional groups. Herein, based on the high selectivity of MIPs and the fluorescence properties of the β-NaYF_4_:Yb^3+^, Er^3+^ upconversion nanoparticles, MIPs with both specificity and fluorescent signals are fabricated to recognize trace sterigmatocystin (ST) with high selectivity and sensitivity. The structure analogue of ST, 1,8-dihydroxyanthraquinone (DT), was employed as the template molecule, acrylamide as the functional monomer, 3-methacryloyloxypropyltrimethoxysilane as the crosslinking agent, and a new molecular imprinting technique of non-aqueous sol-gel method is used to synthesize a molecularly imprinted material with high selectivity to ST. Under optimal conditions, the fluorescence enhancement of fluorescent MIPs increased as the concentration of ST increased. In the range of 0.05–1.0 mg L^−1^, fluorescence enhancement and the concentration showed a good linear relationship with a detection limit of 0.013 mg L^−1^. Real sample analysis achieved the recoveries of 83.8–88.8% (RSD 5.1%) for rice, 82.1–87.5% (RSD 4.6%) for maize, and 80.6–89.2% (RSD 3.0%) for soybeans, respectively, revealing the feasibility of the developed method.

## 1. Introduction

Originated from the intention to create binding sites in bottom-up synthetic polymers, molecular imprinting technology (MIT) has acted as a powerful tool for preparing materials that can bind to analytes reversibly and selectively in the presence of the interferents, and has provided a promising and advantageous alternative to satisfy the need of analytical methods with high sensitivity and good reliability for food safety inspection [1,2,3,4]. As the new-designed polymeric receptors, molecularly imprinted polymers (MIPs) are typically three-dimensional polymeric networks fabricated by functionalized monomers through template-assisted polymerization or polycondensation processes [5,6,7,8,9,10,11]. Considered as the mimics of natural recognition entities such as antibodies and biological receptors, MIPs possess selective and specific cavities for recognition and binding of the target molecules with respect to size, shape, and functional groups, [12,13,14]. Compared with the conventional receptors, MIPs have exhibited advantageous performance, including high specificity against complex matrix, excellent mechanical and thermal stability, low cost, easy to prepare, and no need for extensive sample preparation and highly skilled personnel. Involvement of MIT into the design of fluorescence probes/sensors strategies produced well-performed nanophosphor-encoded imprinting polymers, combining the advantages of high selectivity of MIPs with high sensitivity of luminescence of functional nanophosphors (e.g., Quantum Dots (QDs) or upconversion nanoparticles). Employing the nanophosphors as the signal-reporters, the composite MIPs materials demonstrated remarkable and flexible quantification performance due to their advantageous surface area, superb optical properties, and the capability of multiplex sensing, and have widely applied in biosensing, food-safety inspection, and environmental monitoring [15,16,17,18,19]. QDs-embedded MIPs probe has been extensively studied and applied, but suffered from high toxicity, poor anti-photobleaching, and high background interference from the sample matrix [17,20,21].

Anti-Stokes nanophosphors, especially the inorganic upconversion nanocrystals with near-infrared excitable luminescence, have evoked increasing interest in the development of innovative sensing strategy [22,23,24,25,26,27]. Upconversion refers to nonlinear optical processes in which the sequential absorption of two or more photons leads to the emission of light at a shorter wavelength (usually in the visible range) than the excitation wavelength (infrared or near infrared) [28,29,30]. The corresponding mechanisms have been fully discussed, mainly based upon the sequential absorption of two or more photons by metastable and long-lived energy states. This sequential absorption leads to the population of a highly excited state from which upconversion emission occurs [31,32]. Typically, inorganic upconversion nanophosphors consist of a crystalline host and a lanthanide-based dopant added in low concentrations, in which the dopant provides luminescent centers while the host lattice provides the matrix to bring these centers into optimal position [24]. The upconversion feature enables the upconversion nanocrystals to be utilized as advantageous nanoprobes for sensing and imaging, along with high quantum yields, narrow emission peaks, long lifetimes, large Stokes shifts, superior photostability, and low toxicity [33,34,35]. Combination of Upconversion Nanoparticles (UCNPs) with MIPs would produce the superior composite functional polymers with excellent performance for sensing and probing.

**Figure 1:**
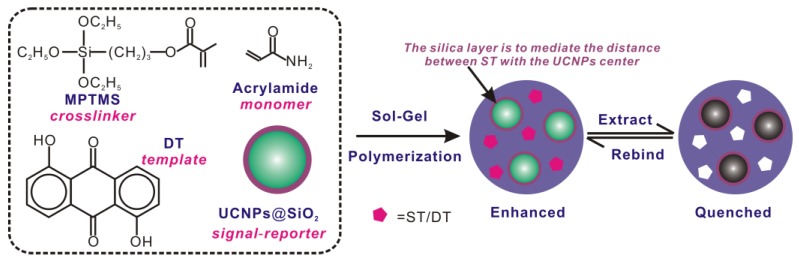
Schematic illustration of upconversion nanophosphor-involved molecularly imprinted fluorescent polymers for sensitive and specific recognition of sterigmatocystin.

Herein, based on the high selectivity of MIPs and the fluorescence properties of the upconversion nanoparticles, MIPs with both specificity and fluorescent signals are fabricated to recognize trace sterigmatocystin (ST) with high selectivity and sensitivity. Sterigmatocystin (ST), a secondary metabolite produced by fungi, appeared as the significant contaminates of grains and feeds, with potential carcinogenic, teratogenic, and mutagenic risks [36,37,38,39,40,41]. The analytical performance of the conventional methods for determination of ST, including high performance liquid chromatography (HPLC) and liquid chromatography-mass spectrometry (LC-MS), was insufficient in terms of speed, simplicity, sensitivity, as well as the specificity against a complex sample matrix [42,43,44,45,46]. In the present work, the hexagonal phase upconversion fluorescent nanoparticles (β-NaYF_4_: Yb^3+^, Er^3+^) were prepared by pyrolysis using yttrium acetate tetrahydrate, ytterbium acetate tetrahydrate, and erbium acetate tetrahydrate and transferred from oil phase to aqueous phase with the aid of Triton X-100. Then, silica coating on the as-prepared UCNPs was performed via the Stöber alkaline hydrolysis to obtain the fluorescent carrier, UCNP@SiO_2_, which was then utilized in the following surface-imprinted polymerization. The structure analogue of ST, 1,8-dihydroxyanthraquinone was employed as the template molecule, acrylamide as the functional monomer, 3-methacryloyloxypropyltrimethoxysilane as the crosslinking agent, and a new molecular imprinting technique of non-aqueous sol-gel method was introduced to synthesize the MIPs material with high specificity to ST (Figure 1). Results of adsorption experiments showed that under optimal conditions, the fluorescence enhancement of fluorescent molecularly imprinted polymers increased as the concentration of ST increased. In the range of 0.05–1.0 mg·L^−1^, fluorescence enhancement of fluorescent imprinted polymers and the concentration showed a good linear relationship with a detection limit of 0.013 mg·L^−1^. In order to verify the practicability of the established method, rice, maize, and soybeans are tested for ST detection in this paper. The standard addition recovery test shows that with addition concentration of rice, maize, and soybeans of 50, 100, and 200 μg·kg^−1^, the recovery rates are 83.8–88.8% (RSD 5.1%), 82.1–87.5% (RSD 4.6%), and 80.6–89.2% (RSD 3.0%) respectively, which demonstrates the feasibility of the established method.

## 2. Materials and Methods

### 2.1. Chemicals and Materials

All chemicals used were at least analytical grade. Ultrapure water (18.2 MΩ·cm) obtained from a WaterPro water purification system (Labconco Corporation, Kansas City, MO, USA) was used throughout. 3-methacryloyloxypropyltrimethoxysilane (MPTMS, >98%), Tetraethyl ortosilicate (TEOS, >98%), yttrium acetate tetrahydrate, ytterbium acetate tetrahydrate, erbium acetate tetrahydrate, 1,8-dihydroxyanthraquinone, acrylamide, NH_3_·H_2_O, Octadecene (OTC), Oleic acid (OA), and 2,2’-Azobisisobutyronitrile (AIBN) were all purchased from Aladdin (Shanghai, China). Triton X-100 was purchased from Sinopharm Chemical Reagent (Beijing, China). Sterigmatocystin, Zearalenone (ZEN), Aflatoxin B1 (AFT B1), Aflatoxin B2 (AFT B2), Aflatoxin M1 (AFT M1), microcystin-leucine-arginine (MC-LR), ochratoxin A (OTA), and vomitoxin (DON) were all purchased from Sigma-Aldrich (St Louis, MO, USA). Methanol, ethanol, acetonitrile, acetone, chloroform, and n-hexane were all obtained from Guangfu Fine Chemical Research Institute (Tianjin, China). All the biomolecules, including the proteins and enzymes, were from Newprobe Biotechnology Co. Ltd. (Beijing, China). All the glassware was cleaned with aqua regia (HCl:HNO_3_ = 3:1, *v*/*v*) and thoroughly rinsed with ultrapure water before use.

### 2.2. Instrumentation

The luminescence spectra of UCNPs measurements were performed on an F-4500 spectrofluorometer (Hitachi, Tokyo, Japan) equipped with a plotter unit and a quartz cell (1 cm × 1 cm) and connected with an external 980-nm diode laser (1 w, continuous wave with 1-m fiber, Beijing Viasho Technology Co., Beijing, China). The morphology and microstructure of the persistent luminescence nanoparticles were characterized by transmission electron microscopy (TEM) on a JEOL-100CX-II (JEOL, Tokyo, Japan) microscope operating at a 200 kV accelerating voltage. The samples for TEM were obtained by drying sample droplets from water dispersion onto a 300-mesh Cu grid coated with a carbon film, which was then allowed to dry prior to imaging. The X-ray diffraction (XRD) spectra were collected on a Rigaku D/max-2500 X-ray diffractometer (Rigaku, Tokyo, Japan) with Cu KR radiation. The scanning electron microscopy (SEM) images were recorded on a LEO 1530VP (LEO, Berlin, Germany) microscope at 10.0 kV. Fourier transform-infrared spectra (FT-IR) (4000–800 cm^−1^) were measured on a TENSOR 27 spectrometer (Bruker, Berlin, Germany) with pure KBr as background. Dynamic Light Scattering (DLS) analysis was performed on a Malvern Zetasizer 3000HSa (He−Ne laser, λ = 632.8 nm).

### 2.3. Preparation of UCNPs

The β-NaYF_4_: Yb^3+^, Er^3+^ UCNPs were prepared referring to the reported methods with some modifications [47]. In a typical assay, 263.72 mg (0.78 mmol) Y(CH_3_COO)_3_·4H_2_O, 84.45 mg (0.20 mmol) Yb(CH_3_COO)_3_·4H_2_O, and 6.9 mg (0.02 mmol) Er(CH_3_COO)_3_·4H_2_O were dissolved in a mixture of 6 mL of OA and 17 mL of OTC, and heated to 110 °C under vigorous stirring to thoroughly eliminate the water from the chemicals. Then the temperature was slowly increased to 160 °C under He atmosphere to make the salts completely dissolved to obtain the uniform light yellow solutions. After the mixture cooled to the room temperature, 10 mL methanol containing 0.1 g (0.20 mmol) NaOH and 0.1482 g (4.0 mmol) NH_4_F was slowly added into the above solutions under stirring to get a milk white mixture and proceeded to heating at 65 °C for 30 min to eliminate the methanol followed by another 1.5 h at 120 °C heating under vacuum to eliminate the water and O_2_. The reaction mixture was finally heated to 350 °C for 1 h and cooled down to room temperature to produce the white precipitate as the UCNPs nanomaterials. The products were washed with ethanol with three round of centrifugation (1000 rpm, 10 min), and dried under vacuum before stored in dark.

To transform the above-obtained hydrophobic UCNPs to hydrophilic UCNPs nanoparticles, 0.1 g of hydrophobic UCNPs powder was mixed with 20 mL of Triton X-100 into a 250 mL flask, and kept under ultrasonication for 30 min. Then 80 mL of water was added into the mixture followed by vigorous stirring for 6 h. The obtained hydrophilic UCNPs nanoparticles were thoroughly washed with water via centrifugation (6000 rpm, 30 min, three times), and dried under vacuum before being stored in dark.

### 2.4. Preparation of UCNPs@SiO_2_

The silica coating was performed according to the Stöber method [48]. Briefly, 0.1 g of the above hydrophilic UCNPs was homogenously dispersed in 150 mL of ethanol with the assistance of ultrasonication. Then 40 mL of water and 1 mL of NH_3_·H_2_O were separately added and the mixture was kept stirring at 30 °C for 10 min. After that, 0.06 g of TEOS dissolved in 10 mL of ethanol was dropwise added into the mixture and the reaction was allowed to proceed under vigorous stirring for 6 h. The obtained UCNPs@SiO_2_ nanoprobes were washed with ethanol three times, and dried under vacuum before stored in dark.

### 2.5. Preparation of UCNPs@SiO_2_ MIPs

The UCNPs@SiO_2_ MIPs were prepared via the non-aqueous sol-gel procedure, applying the UCNPs as the emission center, the structure analogue of ST, 1,8-dihydroxyanthraquinone (DT) as the template molecule, acrylamide as the functional monomer, and MPTMS as the crosslinking agent. Typically, 130.7 mg of DT and 150.67 mg of acrylamide were dissolved in 5 mL of the component solvent of chloroform and acetonitrile (4:1, *v*/*v*), and shaken for 30 min. Then 100 mg of as-prepared UCNPs@SiO_2_ was gently added to the above mixture. After 30-min stirring, 745 mg of MPTMS with 0.01 g AIBN as initiator were added under ultrasonication to eliminate the air, and treated with N_2_ purge for 20 min, and sealed. The reaction was allowed to process at 60 °C for 18 h. The products were thoroughly grinded, filtrated through a 200-mesh sieve, and treated by a Soxhlet extractor with 200 mL of acetone and acetonitrile (9:1, *v*/*v*) to remove the template. The obtained MIPs with specific cavities were dried under vacuum and stored in dark. To provide a reference, non-imprinted polymers (NIP) based on UCNPs@SiO_2_ were prepared using the same procedure but without the addition of the template molecule (DT).

### 2.6. Imprinting Procedures

To investigate the optimal adsorption media, 2.0 mg of UCNPs@SiO_2_@MIP materials were weighed into a series of 4-mL tubes, and separately mixed with 3 mL of ST standard solutions prepared by methanol, chloroform, acetonitrile, n-hexane, and acetone, respectively. After being shaken for 7 h at room temperature for thorough adsorption, the upconversion fluorescence (EX 980 nm, EM 540 nm) was measured to assess the adsorption efficiency. Equivalent amounts of UCNPs@SiO_2_@NIP were performed via the same procedure as the control.

To perform the static adsorption assay, 2.0 mg of UCNPs@SiO_2_@MIP materials were weighed into a series of 4 mL tubes, and separately mixed with 3 mL of ST standard solutions (methanol) with concentrations of 0.05, 0.1, 0.2, 0.4, 0.6, 0.8, 1.0, 2.0, 4.0, 10.0 and 20.0 mg·L^−1^, respectively. After gently shaking for 7 h (90 rpm) at room temperature for thorough adsorption, the upconversion fluorescence (EX 980 nm, EM 540 nm) was measured to assessing the adsorption efficiency. Equivalent amounts of UCNPs@SiO_2_@NIP were performed via the same procedure as the control.

To perform the dynamic adsorption assay, 2.0 mg of UCNPs@SiO_2_@MIP materials were weighed into a series of 4-mL tubes, and separately mixed with 3 mL of ST standard solutions (methanol) with concentrations of 1.0 mg·L^−1^, and treated with continuous shaking at room temperature. The upconversion fluorescence (EX 980 nm, EM 540 nm) was measured to assess the adsorption efficiency at the time points of 0.5, 1.0, 1.5, 2.0, 2.5, 3.0, 3.5, 4.0, 4.5, 5, 6, 8 and 10 h. Equivalent amounts of UCNPs@SiO_2_@NIP were performed via the same procedure as thecontrol.

To assess the specificity of the developed UCNPs@SiO_2_@MIP materials, 2.0 mg of UCNPs@SiO_2_@MIP materials were weighed into a series of 4-mL tubes, and separately mixed with 3 mL of interferent standard solutions (methanol) with concentrations of 10.0 mg·L^−1^. After shaking for 7 h at room temperature for thorough adsorption, the upconversion fluorescence (EX 980 nm, EM 540 nm) was measured to assess the adsorption efficiency, and compared with that of ST. Equivalent amounts of UCNPs@SiO_2_@NIP were performed via the same procedure as the control.

## 3. Results and Discussion

### 3.1. Preparation and Characterization of the UCNPs@SiO_2_@MIP Materials

**Figure 2:**
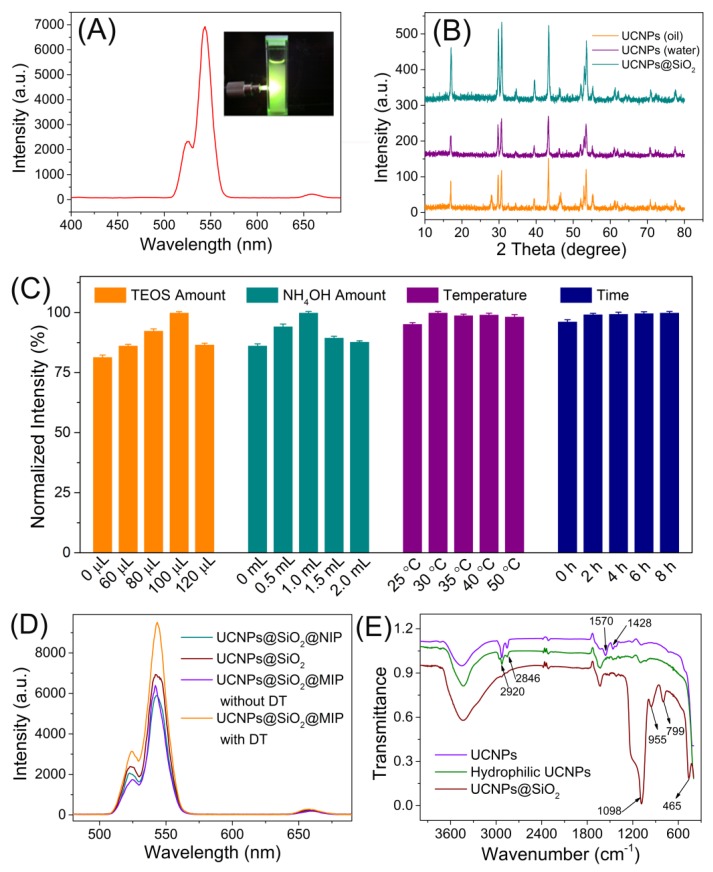
(**A**) Upconversion luminescence spectra of the as-prepared β-NaYF_4_: Yb^3+^, Er^3+^ nanocrystals with inset as the typical photograph of the UCNPs under excitation; (**B**) The XRD patterns of the UCNPs powders: hydrophobic UCNPs (green), hydrophilic UCNPs (purple), and UCNPs@SiO_2_ (orange); (**C**) Optimization of the silica coating procedures: Tetraethylortosilicate (TEOS) amount (orange), NH_4_OH amount (teal), reaction temperature (purple), and reaction time (blue). (**D**) Comparison of the fluorescence intensity of different polymeric materials; (**E**) The FT-IR spectra of the UCNPs and UCNPs@SiO_2_.

Compared with the conventional fluorescence probes, like semi-conductor QDs and organic dyes, UCNPs possess the capability of avoiding auto-fluorescence and have long lifetimes and low photobleaching, thus are fit to act as the signal-reporter for the determination of target molecules against complex sample matrix. In the present work, β-NaYF_4_: Yb^3+^, Er^3+^ upconversion nanoparticles were selected as the emission center, which are typical colloidal nanocrystals of hexagonal-phase, lanthanide-doped, rare-earth fluorides with high upconversion luminescence efficiency. The cost-effective hydrothermal procedure that can generate nanocrystals with well-controlled morphology was introduced for the preparation of UCNPs. The nanostructured β-NaYF_4_: Yb^3+^, Er^3+^ were successfully synthesized via that facile hydrothermal approach, using oleic acid as a stabilizing agent, and NH_4_F and metal acetates as precursors, in the temperature range of 110–160 °C under basic conditions. The visible luminescence spectra of the obtained β-NaYF_4_ codoped with Er^3+^ and Yb^3+^ displayed two emission bands under infrared excitation (980 nm), which can be assigned to the 4f-4f transitions of the Er^3+^ ions. A dominant green emission originating from ^2^H_11/2_, ^4^S_3/2_→^4^I_15/2_ transition, while the weak red emission at 660 nm was attributed to the ^4^F_9/2_→^4^I_15/2_ transition. X-ray diffraction (XRD) patterns indicated that the nanostructures obtained after heating consist of pure β-NaYF_4_ (Figure 2). The obtained hydrophobic UCNPs were treated with the surface modifier Triton X-100 to transform to hydrophilic nanoparticles to favor the following silica coating. 

Silica coating on the UCNPs surface was performed via the Stöber method to generate the UCNPs@SiO_2_ nanostructures, in which the silica layer acted as the protection of UCNPs against possible damage to the nanostructure and the spacer between UCNPs and DT to mediate their spectral interaction by providing beneficial distance. XRD analysis indicated the surface modification barely affect the crystalline nanostructures of UCNPs (Figure 2B). FT-IR spectra of UCNPs and UCNPs@SiO_2_ showed the peaks of 1570 and 1428 cm^−1^ belonged to the asymmetric and symmetric stretching vibrations of the carboxylic group, while peaks of 2920 and 2846 cm^−1^ were assigned to the asymmetric and symmetric stretching vibrations, respectively, of a methylene group that existed in the long alkyl chain of the oleic acid molecule. Peaks at 465, 799, 955 and 1098 cm^−1^ represented the Si–O–Si bending vibration, Si–OH stretching vibration, and Si–O–Si stretching vibration, respectively. All the above confirmed the successful synthesis of UCNPs@SiO_2_ nanostructures (Figure 2E).

**Figure 3:**
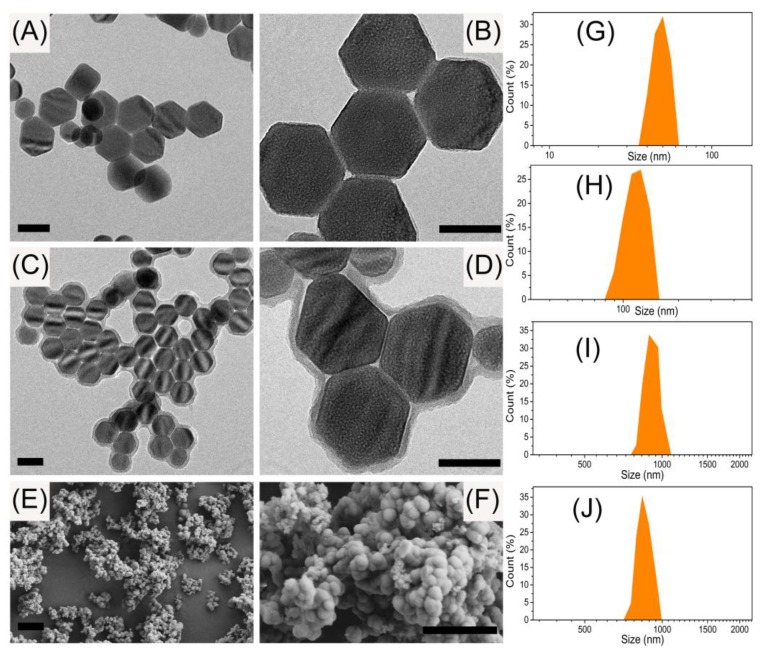
(**A**,**B**) The typical HRTEM photographs of the as-prepared β-NaYF_4_: Yb^3+^, Er^3+^ upconversion nanocrystals; (**C**,**D**) The typical HRTEM photographs of the UCNPs@SiO_2_ nanocrystals; (**E**,**F**) The typical SEM images of the obtained UCNPs@SiO_2_@MIP polymeric materials. The scale bars represent 50 nm (**A**–**D**) and 2 μm (**E**,**F**), respectively; The size distribution of UCNPs (**G**), UCNPs@SiO_2_ (**H**), MIPs (**I**), and NIPs (**J**) obtained via Dynamic Light Scattering (DLS).

The experimental conditions of the silica coating process that would possibly affect the resultant MIP materials performance have been extensively investigated, including the TEOS amount, NH_4_OH amount, reaction time, and temperature. Because the silica coating layer thickness closely depended on the TEOS amount, the fluorescence of the final MIPs increased as the TEOS amount increased. Using 100 μL of TEOS produced the strongest fluorescence, indicating the resultant silica layer provided the optimal spacer for the interaction between UCNPs with DT molecules embedded inside the MIPs as the templates. The optimal amount of NH_4_OH, which decided the silane hydrolysis rate, was assessed as 1.0 mL, resulting the best fluorescence performance of the as-prepared MIPs. The reaction time and temperature showed no significant influence on the MIPs fluorescence. Thus the optimal experimental conditions are as follows: 100 μL of TEOS; 1.0 mL of NH_4_OH, 30 °C; 6 h. TEM and SEM characterizations revealed the as-prepared UCNPs, UCNPs@SiO_2_, and UCNPs@SiO_2_@MIPs possessed sizes of 51 ± 3 nm, 105 ± 8 nm, and 808 ± 19 nm, respectively, with narrow size distribution (Figure 3). When a 980 nm diode laser with a 2 W output was focused on the UCNPs@MIP, strong upconversion luminescence was visible to the naked eye in the dark (Figure 2A).

### 3.2. MIPs Recognition of ST

The UCNPs@SiO_2_ MIPs were prepared via the non-aqueous sol-gel procedure, applying the UCNPs as the emission center, the structure analogue of ST, 1,8-dihydroxyanthraquinone (DT) as the template molecule, acrylamide as the functional monomer, and MPTMS as the crosslinking agent. As shown in Figure 2D, silica coating had little effect on the fluorescence of UCNPs, and the following polymerization of imprinting materials produced remarkable enhancement of the UCNPs luminescence. Removal of the template, DT, resulted in the turn-off of the fluorescence, and specific cavities for ST. 

It was well-demonstrated and reported that the luminescence of UCNPs could be regulated via the presence of an energy acceptor or an electron acceptor nearby [49,50,51,52]. One feasible way is to utilize the plasmonic nanoparticles or nanoshell to enhance the luminescence of UCNPs via the energy transfer while the UCNPs and metal nanostructures are close and possess certain spectra overlap [49]. Because there is barely any overlap between emission spectra of UCNPs with the absorption spectra of DT and ST, the mechanism of the luminescence enhancement may not derive from the energy transfer process. It was also reported that the luminescence of UCNPs could be affected by the entity nearby via the photoinduced electron transfer (PET) process, through which the luminescence was able to be enhanced to some extent by organic molecules with conjugated structures [53,54]. In the present work, DT and ST both possess the typical conjugated structures. When the UCNPs were excited by 980 nm laser, the PET process happened between DT/ST with UCNPs to produce the luminescence enhancement. In addition, as reported in the literature [49,54], the luminescence modulation effect significantly depends on the distance between UCNPs with molecules, thus silica coating on the UCNPs was performed first to act as the spacer to regulate the distance between the nanoparticle with ST/DT. It was also found the fluorescence of UCNPs showed little response to the presence of DT or ST without the silica layer spacing. Using the UCNPs without silica coating for imprinting ST failed for the determination of the target (Supporting Information, Figure S1).

The MIP formation process have been carefully optimized. The ratio of template, monomer, and cross-linker is the basic experimental condition of the MIPs fabrication. Increase of monomer amount would increase the interaction chance with DT, generating more effective recognition sites inside the MIPs, while excessive monomers would directly bind with the cross-linker to produce the non-specific cavities, thus reduce the imprinting efficiency. The appropriate amount of cross-linker benefited the formation of a well-organized polymeric structure and increased the adsorption rate. Insufficient cross-linker would lead to the low crosslinking density, looseness of MIPs structure, and poor recognition ability, while the excess cross-linker would result in the self-polymerization, inhomogeneous structure, and difficulty in removal of the template. Performance of different ratios and reaction time and temperature have been fully evaluated according to the imprinting factors of the resultant MIPs materials. The imprinting factor (IF) was calculated via the ratio of adsorption capability of MIPs (Q_MIP_) to that of NIPs (Q_NIP_) as: IF = Q_MIP_/Q_NIP_.

**Figure 4:**
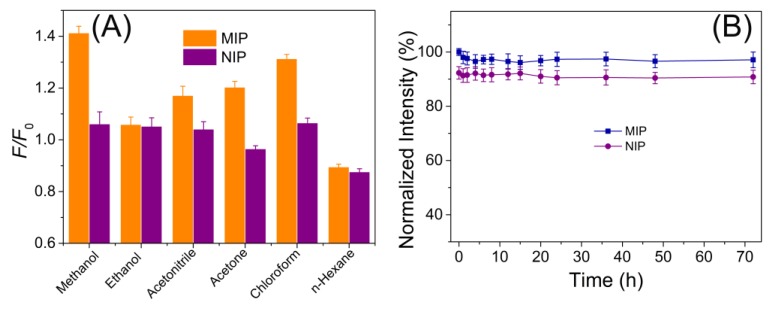
(**A**) The effect of adsorption media on the specific recognition performance of MIP and NIP; (**B**) The fluorescence stability of the MIP and NIP materials.

Results in Table 1 and Table 2 indicated that the optimal ratio of template/monomer/cross-linker was 1:4:6 and the optimal reaction condition was 60 °C and 18 h. Furthermore, the effect of reaction media for imprinting ST was assessed by the comparison of the extent of fluorescence enhancement of ST to MIPs in different solvent, including methanol, chloroform, acetonitrile, n-hexane, and acetone. When using methanol, the MIPs gave the highest enhancement response, while the corresponding NIPs gave the relatively low response (Figure 4A), thus methanol was chosen as the ideal media for imprinting. In addition, the as-prepared MIPs and NIPs both demonstrated excellent fluorescence stability (Figure 4B).

### 3.3. MIPs Performance for ST

**Figure 5:**
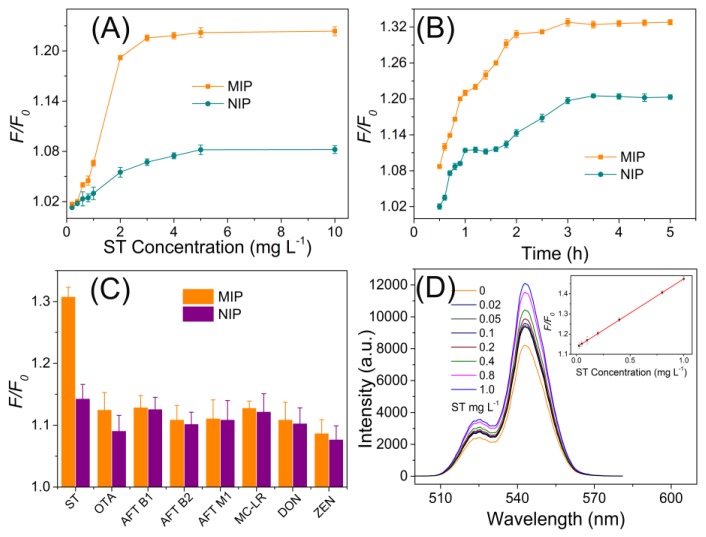
(**A**) Comparison of the static adsorption properties of MIP and NIP materials; (**B**) Comparison of the dynamic adsorption properties of MIP and NIP materials; (**C**) The evaluation of the specificity of the developed UCNPs-involved MIP polymeric materials; (**D**) The linear response of the developed MIP probe to ST.

Generally, the fluorescence response depends on the adsorptive affinity of the particles with the template. In the case of the MIP coated UCNPs, the fluorescence response was mainly achieved by the affinity of the imprinted cavities to the template, due to specific interactions. To evaluate the specific imprinting capability of MIPs to ST, static adsorption (Figure 5A) and dynamic adsorption (Figure 5B) assays were carried out. In the static adsorption, the fluorescence response increased as the ST concentration increased. When the ST concentration up to 4 mg·L^−1^, the response tended to achieve a balance, indicating the MIPs recognition of ST got saturated. Meanwhile, the corresponding NIPs response showed the similar trend, but much lower than that of MIPs. In the dynamic adsorption, the MIPs and NIPs were separately incubated with ST (1.0 mg·L^−1^), and the fluorescence was continuously monitored for 5 h. Almost 50% of the binding was obtained within a short shaking period of 1 h, and the fluorescence response increased to the top within 2 h. It is implied that UCNPs@MIP have a faster template loading rate compared with NIPs.

To test the specificity of the developed UCNPs-embedded MIPs probes for ST, the common food toxins (5.0 mg·L^−1^), including OTA, AFT B1, AFT B2, AFT M1, MC-LR, DON, and ZEN, were chosen as the interferents. Thanks to the well-constructed imprinting polymers, the UCNPs-involved showed insignificant response to the interferents, compared with that of the target ST (0.5 mg·L^−1^) (Figure 5C). The proposed fluorescent MIPs method gave a linear range of 0.02–1.0 mg·L^−1^ (5–250 μg·kg^−1^ for cereal samples) with a detection limit (3s) of 0.013 mg·L^−1^ for the detection of ST. The precision (relative standard deviation, RSD) for eleven replicate detections of 0.5 mg·L^−1^ ST was 2.5%. All the above results demonstrated the high sensitivity and specificity of the developed fluorescent MIPs to ST. The maximum limits of sterigmatocystin in agro-products are regulated as 5–20 µg·kg^−1^ in Czech Republic and Slovakia, the California Department of Health Services set the maximum intake level of 8 μg/kg body weight/day for a 70 kg adult, and the regulatory sterigmatocystin level of below 25 μg·kg^−1^ is accepted in China. Thus the sensitivity of the developed method could satisfy the need of regulation [39]. 

The developed MIP method has been compared with the previously-reported methods for ST determination, in terms of sensitivity and linear range. (Table 3) Our method showed comparable performance with the optical methods and insufficient sensitivity to the LC-MS and ELISA methods, but was time- and labor-effective.

### 3.4. Real Sample Analysis

To demonstrate the applicability of the developed UCNPs-involved MIPs for real sample analysis, it was employed for specific quantification of ST in cereal samples. Before analyzing, the cereal samples were thoroughly grinded, treated with 60-mesh filters, and extracted with methanol. The obtained cereal sample solutions were analyzed by the fluorescent MIPs using standard sample recovery. As shown in Table 4, the obtained recoveries of the spiked samples were in the range of 83.3–89.5% for rice, 82.1–91.0% for maize, and 80.6–89.2% for soybean, respectively. The ST standard was spiked into the cereal samples after grinding, then gave sample preparation before analysis. The possible reason of the recovery being less than 100% is the unavoidable amount loss during the sample preparation, like the extraction or filtration.

## 4. Conclusions

In conclusion, a novel nanophosphor–involved MIPs polymeric materials, employing β-NaYF_4_: Yb^3+^, Er^3+^ upconversion nanoparticles as the emission centers, have been fabricated for determination of ST with excellent specificity and sensitivity. Silica coating on the UCNPs favored the turn-on fluorescence recognition of targeted ST molecules, and the non-aqueous sol-gel synthetic strategy has produced upconversion nanoparticles with highly stable anti-stokes luminescence. The developed UCNPs-based MIPs were demonstrated practical for real sample analysis, and accurate and sensitive quantification of ST in cereal samples was accomplished. Upconversion nanophosphor-involved MIPs have been qualified as a promising platform for robust and efficient nanoprobes for specific molecule recognition and rapid sensing in complex samples.

## Figures and Tables

**Table 1:** The optimization of template/monomer/cross-linker ratios for the polymerization.

Polymer	DT (mmol)	AM (mmol)		MPTMS (mmol)	AIBN (mg)	Imprinting factor
MIP1/NIP1	1.0	3.0	6.0		10.0	1.4506
MIP2/NIP2	1.0	3.0	8.0		10.0	1.2874
MIP3/NIP3	1.0	4.0	4.0		10.0	-
MIP4/NIP4	1.0	4.0	6.0		10.0	3.4876
MIP5/NIP5	1.0	4.0	8.0		10.0	3.2404
MIP6/NIP6	1.0	5.0	4.0		10.0	2.8362
MIP7/NIP7	1.0	5.0	6.0		10.0	2.7018
MIP8/NIP8	1.0	5.0	8.0		10.0	1.9183

**Table 2:** The optimization of reaction temperature and time for the polymerization.

Polymer	DT (mmol)	Temperature (°C)	Time (h)	AIBN (mg)	Imprinting factor
MIP10/NIP10	1.0	50	18	10.0	1.1238
MIP3/NIP3	1.0	60	18	10.0	3.4876
MIP15/NIP15	1.0	60	12	10.0	-
MIP16/NIP16	1.0	60	24	10.0	-
MIP17/NIP17	1.0	70	18	10.0	1.0782

**Table 3:** Comparison of the developed UCNPs-involved MIP method with the previously-reported methods for ST determination.

Method	Detection limit (μg·L^−1^)	Linear range (μg·L^−1^)	Reference
ELISA	0.36	-	[39]
Fluorescence	19	50–2000	[42]
LC-MS	0.025	*NA*	[43]
GC-MS	3	10–150	[44]
LC-MS	3	-	[45]
LC-MS	3	-	[46]
Fluorescence	13	20–1000	This work

**Table 4:** Application of the developed MIP probe for the determination of ST in cereal samples (Mean ± SD).

Samples	Spiked (μg·kg^−1^)	FL intensity		Determined (μg·kg−1)	Recovery (%)	RSD (%)
		Initial	Sampling			
Rice	10	8120	9321	8.6 ± 0.2	86.0	2.3
	20	8120	9378	17.9 ± 0.5	89.5	2.8
	50	8120	9419	44.4 ± 0.8	88.8	1.8
	100	8120	9581	83.3.2 ± 1.3	83.3	1.6
	200	8120	9987	168.0 ± 2.9	84.0	1.7
Maize	10	7856	9018	8.8 ± 0.2	88.0	2.3
	20	7856	9087	18.2 ± 0.4	91.0	2.2
	50	7856	9127	42.8 ± 3.7	85.6	4.0
	100	7856	9301	82.1 ± 1.0	82.1	1.2
	200	7856	9741	182.6 ± 3.1	87.8	1.7
Soybean	10	7980	9158	8.8 ± 0.3	88.0	3.4
	20	7980	9207	17.7 ± 0.4	88.5	2.2
	50	7980	9261	40.3 ± 2.4	80.6	5.9
	100	7980	9466	86.3 ± 1.8	86.3	2.1
	200	7980	9879	178.4 ± 2.3	89.2	1.3

## Supplementary Materials

The following are available online at www.mdpi.com/2073-4360/9/7/299/s1, Figure S1: Comparison of UCNPs intensity for interaction with DT/ST with or without the silica coating.
