# Supplementary material for: Upconversion Nanophosphor-Involved Molecularly Imprinted Fluorescent Polymers for Sensitive and Specific Recognition of Sterigmatocystin
Source: Polymers (Basel). 2017 Jul 22;9(7):299. doi: 10.3390/polym9070299 (PMC6432482; doi:10.3390/polym9070299)
Supplement: Supplementary file 1 [file polymers-09-00299-s001.pdf]

Supporting Information

Upconversion Nanophosphor-Involved Molecularly Imprinted Fluorescent Polymers for Sensitive and Specific Recognition of Sterigmatocystin

Jing-Min Liu[1,2], Feng-Zhen Cao[3], Guo-Zhen Fang[3], and Shuo Wang[1,2]*

[1] Beijing Advanced Innovation Center for Food Nutrition and Human Health, Beijing Technology & Business University (BTBU), Beijing, 100048, China

[2] School of Medicine, Nankai University, Tianjin, 300071, China

[3] Key Laboratory of Food Nutrition and Safety, Ministry of Education, Tianjin University of Science and Technology, Tianjin, 300457, China

* Correspondence: wangshuo@nankai.edu.cn; Tel.: +86-22-60912490

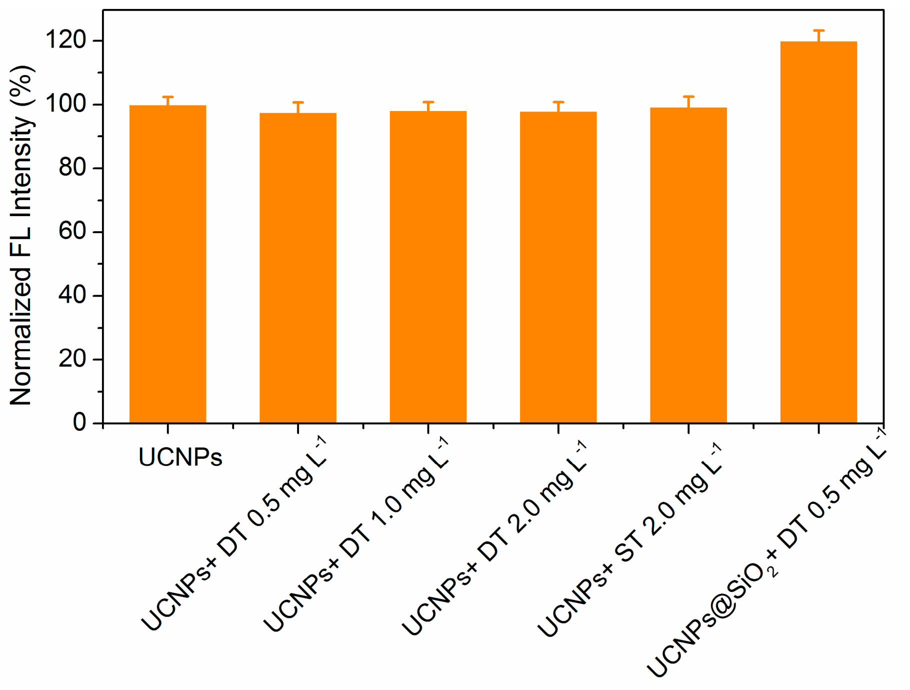

Figure. S1 Comparison of UCNPs intensity for interaction with DT/ST with or without the silica coating.
